# Rehabilitation training improves nerve injuries by affecting Notch1 and SYN

**DOI:** 10.1515/med-2020-0045

**Published:** 2020-05-15

**Authors:** Mao Jing, Yang Yi, Zhang Jinniu, Kan Xiuli, Wu Jianxian

**Affiliations:** Department of Rehabilitation Medicine, The second hospital of Anhui Medical University, Hefei, Anhui, China, 230601; Department of Pathology, Basic Medical College, Anhui Medical University, Hefei, Anhui, China, 230032

**Keywords:** chronic cerebral ischemia, Notch1, rehabilitation training, SYN

## Abstract

**Objective:**

The aim of this study was to investigate the effects of rehabilitation training on Notch1 and synaptophysin (SYN) levels in brain tissues of rats with chronic cerebral ischemia.

**Methods:**

Eighty-one male Sprague-Dawley rats were divided into nine groups: three Sham groups, three Model groups, and three training groups. There were nine rats in each group. At different time points, the apoptosis cell rate was analyzed by the TUNEL assay, and the expression levels of Notch1 and SYN in brain tissues were analyzed by immunohistochemical staining and RT-qPCR assay.

**Results:**

The apoptosis cell rate of training groups was significantly higher on day 28 (*P* < 0.05). The protein and mRNA levels of both Noth1 and SYN in training groups were significantly higher on day 28 (*P* < 0.05).

**Conclusion:**

Rehabilitation training could improve nerve cell apoptosis by increasing the expression of both Notch1 and SYN.

## 1 Introduction

Chronic cerebral ischemia (CCI) is an important factor in the occurrence and development of vascular cognitive impairment. If not treated and controlled on time, this condition may lead to persistent or progressive cognitive and neurological dysfunction [1,2]. Early effective intervention and treatment of CCI patients can improve the cognitive function and will be of great significance to patients’ quality of life and daily activities. The Notch pathway influences the growth and development of the nervous system, as well as the axonal and dendritic growth [3]. The Notch signaling pathway directly affects learning and memory functions and plays a major role in the formation of brain memory in adult mice [4]. Synaptophysin (SYN) can well reflect the synaptic plasticity of the nervous system against ischemic injuries. As an indicator for measurement of synaptic density and synaptic remodeling after cerebral ischemia, it is an important marker of synaptic re-establishment. CCI-induced learning and memory dysfunction may be related to synaptic abnormalities in the hippocampus and dentate gyrus [5]. Many studies have established the effects of exercises on cerebral ischemia, including enhanced survival, reduced nerve injuries, improved blood–brain barrier dysfunction, and supported neurological and vascular integrity [6,7,8,9,10]. In this study, a perpetual ligation of the bilateral common carotid arteries was performed in an attempt to explore the possible mechanisms of Notchl and SYN in the rehabilitation training that was intended to improve learning and memory. This would provide a theoretical basis for the clinical use of rehabilitation training to improve the learning and memory functions.

## 2 Materials and methods

### 2.1 Laboratory animals and grouping

Specific-pathogen free Sprague-Dawley rats (*n* = 81, male, 250–300 g) were obtained from Hunan Slac Jingda Laboratory Animal Co., Ltd. All rats were conditioned in an independent ventilated cage at the Animal Center for 1 week. The rats were exposed to circadian lighting and provided with free access to drinking water and feed at a constant temperature of 21 (±2)℃. After 1 week of conditioning, the rats were divided into the rehabilitation training (RT) group, the Sham group, and the Model group. Based on the time period, each group was divided into three subgroups as days 7, 14, and 28, respectively, with nine rats per subgroup.


**Ethical approval:** The research related to animal use has been complied with all the relevant national regulations and institutional policies for the care and use of animals.

### 2.2 Instruments and reagents

The instruments used in this study are as follows: BX51 microscope (Olympus, Japan), XTL-600 surgical microscope for animals (Shanghai Tocan Bio-technology Co., Ltd, China), BM-IX biological tissue embedding station (Leica, Germany), and TS-12A automatic dehydration machine (Xiaogan Hongye Medical Instrument Co., Ltd, China). The materials used in this study are as follows: Notchl, SYN, and β-actin antibody from Wuhan Boster Biological Technology Ltd; hematoxylin staining solution from Beyotime Institute of Biotechnology; and RIPA lysate and developer and fixer kit, and TUNEL Assay Kit purchased from Nanjing KeyGen Biotech Co., Ltd.

### 2.3 Model preparation

All rats were fasted for 12 h and water-deprived for 4 h before surgery. After weighing, anesthesia was performed by intraperitoneal injection of chloral hydrate (0.3 to 0.35 ml/100 g). The rats were fixed in the supine position, and the incision site on the front of the neck was shaved. After routine local disinfection with iodophor, a no. 7 blade was used to make an incision about 2 cm long in the midline of the neck. Hemostatic forceps were used to slowly separate the subcutaneous tissues. After the bilateral sternocleidomastoid was revealed, a glass-dissecting needle was used to isolate the bilateral common carotid arteries. Care should be taken at this point, so as to avoid touching the vagus nerve and causing damages. After the common carotid artery was separated and ligated with surgical suture, close observation should be made to detect whether the rats’ breathing remained normal or whether there was convulsion, thereby determining whether the vagus nerve was mistakenly ligated. After a double check, the blood vessels were cut from the middle of the ligation to completely block the blood flow of the common carotid artery, and the incision was cleaned with sterile saline. The wound was examined, and the incision was sutured once there was no significant bleeding. For the Sham group, the common carotid artery was only isolated without ligation. The vagus nerve should be well protected during the operation, the respiratory changes of the rats should be closely observed, aseptic operation should be carried out, and the rectal temperature of the rats should be kept at about 36.5℃. After the rats were awakened, they were returned to the independent ventilated cage for feeding. After surgery, intraperitoneal injection of penicillin was performed at 2,00,000 U/kg/day for three consecutive days to prevent postoperative infection.

### 2.4 Rehabilitation training

Enriched rehabilitation training exercises were designed for treatment [11]. Enriched rehabilitation training included the enrichment of environment and the exercise therapy 3 days after successful modeling. The enrichment of environment required the regular replacement of the items in the cage of the RT group rats, variably placing the ping-pong ball, turntable, pipe, staircase, rattle, and ladder, and exposure to different sound and light stimulations for 2 h, once a day. The environment was changed twice a week, and the treatment was continuously performed until days 7, 14, and 28 after the successful modeling. The training therapy consisted of the following exercises: balance beam training – a square wooden rod of 170 cm in length and 2 cm in width was placed at a height of 7 cm above the ground. It was used as a balance beam for the rats to walk on it and get trained for 10 min every day. Rotating bar training: the end of a wooden rod of 150 cm in length and 4.5 cm in diameter was fixed on a 3 rpm rotator, which alternately rotated to one side and the other. The rats could grasp, rotate, and walk on the rotating rod for 10 min every day, and the therapy was continuously carried out until days 7, 14, and 28 after the successful modeling.

### 2.5 Apoptosis detection by TUNEL assay

Five rats were selected from each group and killed by decapitation at difference time points (days 7, 14, and 28). The paraffin-embedded sections of the hippocampus tissues were prepared. The brain tissue sections were placed in dimethylbenzene twice, for 5 min each time. The samples were soaked in ethanol with concentration gradients of 100%, 95%, 90%, 80%, and 70%, for 3 min in each concentration gradient. Then, the samples were rinsed with phosphate buffered saline (PBS) twice, for 4 min each time, and were incubated with proteinase K solution for 30 min at 37℃. After that, the sections were washed with distilled water four times, for 3 min each time. The terminal deoxynucleotidyl transferase (TdT) buffer was added dropwise onto the sections, which were then incubated for 5 min at room temperature. Adding 50 µL TdT buffer in a wet box and incubated for 1 h at 37℃. An enzyme reaction solution containing no TdT was added to the negative control group. The sections were placed in Tunel buffer. Stop buffer and wash buffer were added into the staining tank. After incubation for 30 min at 37℃, the tissue sections were washed with PBS, added with the peroxidase-labeled antibody dropwise, placing in a wet box, and incubated again for 30 min at 37℃. After washing with PBS dropwise, diaminobenzidine (DAB) was added. After counterstaining with hematoxylin, the sections were rinsed with tap water, dehydrated using concentration gradient of ethanol, and made transparent with dimethylbenzene twice.

### 2.6 Immunohistochemistry

The remaining five rats in each group were anesthetized, perfused with 4% paraformaldehyde, and immediately decapitated to remove the brain. Half of the removed tissue was fixed in paraformaldehyde and the other half was refrigerated at −80℃ for later use. The tissue placed in paraformaldehyde was routinely dehydrated, made transparent, waxed, and embedded in paraffin, so as to consecutively make coronal sections of 5 µm in thickness. These sections were prepared for immunohistochemistry (IHC) of Notch1 protein and SYN. The paraffin sections were baked in an electric heating incubator at 60℃ for 1 h, dewaxed, and hydrated. In order to block the activity of endogenous peroxide, the sections were incubated in 3% H_2_O_2_ for 10 min at room temperature and washed with PBS for three times to repair the antigen. Fifty microliters of rabbit anti-rat Notchl antibody diluted to 1:60 were added dropwise onto the sections as the primary antibody, with mouse anti-rat SYN antibody diluted to 1:200 as the secondary antibody; the negative control group was added with PBS instead of primary antibody at 4℃ overnight. The sections were washed with PBS three times, for about 5 min each time. Fifty microliters of secondary antibody were added onto the sections, which were then incubated in a wet box for 20 min and rinsed three times with PBS for 5 min. DAB coloring agent was used to develop color, and distilled water was used to stop the reaction. The sections were rinsed with tap water for 10 min and counterstained with hematoxylin for 1 min. Hydrochloric acid alcohol (0.1%) was used for color separation, rinsing, and re-bluing. After microscopic observation of the degree of nuclear staining, the sections were dehydrated, made transparent, and sealed. Image Pro Plus 6.0 software was used to analyze. By subtracting the cumulative optical density of the blank area from that of Notchl and SYN proteins in the subfield CA1 of the hippocampus of the rats in a ×200 field of view, the expressions of Notchl and SYN in the subfield CA1 of the hippocampus at different time points were quantized for the rats in different groups.

### 2.7 RT-qPCR detection

The brain tissues frozen at −80℃ were cut into pieces and ground in a mortar added with liquid nitrogen. Once the samples were ground into powder, 80 mg of powder was taken into the Eppendorf (EP) tube and 1 ml of Trizol lysate was added to mix, and then placed at room temperature for lysing for 5 min. Then, 200 µL of chloroform was added into the EP tube, which was then shaken violently for 15 s and centrifuged at 10,000 rpm for 5 min at 4℃. The supernatant aqueous layer was drawn into a new EP tube, and 500 µL of isopropanol was used to extract the RNA. The tube was gently inverted up and down ten times, allowed to stand at room temperature for 10 min, and centrifuged at 10,000 rpm for 10 min. The supernatant was discarded, and 75% ethanol was added to suspend the RNA precipitation. After the solution was centrifuged at 10,000 rpm for 15 min at 4℃, the supernatant was discarded. The EP tube was placed on a clean bench, air dried, and added with diethylpyrocarbonate to suspend the water. The concentration of the extracted RNA was determined by using an ultraviolet spectrophotometer. The cDNA of miR-181a was synthesized by reverse transcription, as per the instructions of the reverse transcription kit. The gene expression levels of Notch1 and SYN were detected by quantitative reverse transcription polymerase chain reaction (RT-qPCR).

### 2.8 Statistical analysis

The data were analyzed by SPSS 19.0 and shown as mean ± standard deviation. The results of one-way analysis of variance were used to analyze the experimental results, and *P* < 0.05 was considered significantly different. The Dunnett-*t* test was used for the pairwise comparison. The results were all plotted with Graphpad Prism 6.

## 3 Results

### 3.1 Effect of rehabilitation training on brain tissue damage

**Figure 1:**
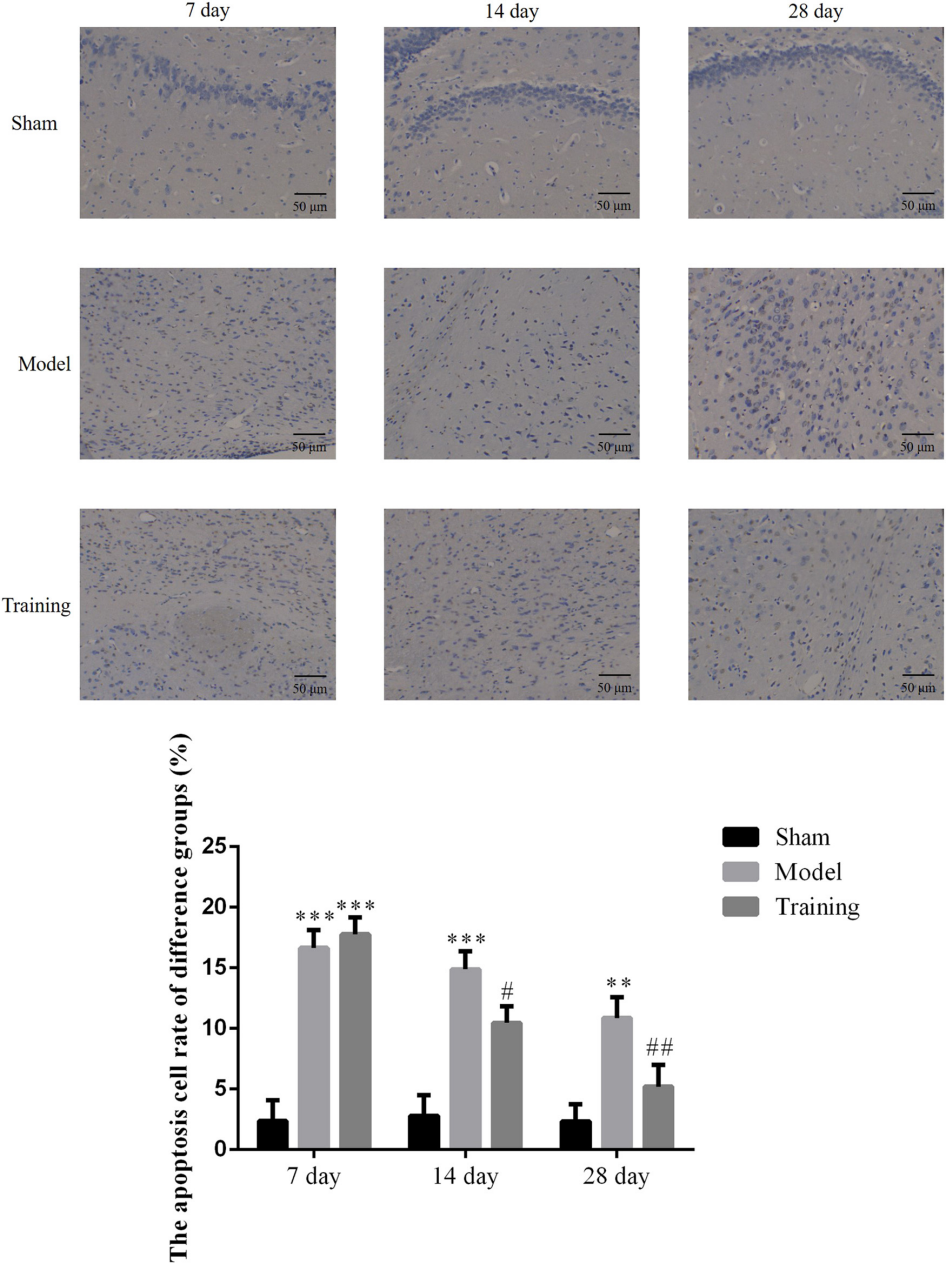
The apoptosis cell rate of different groups by TUNEL assay (×200). Sham: Sham group; Model: nerve injuries in CCI rat model; Training: nerve injuries in CCI model rats that were treated with rehabilitation training exercises; **P < 0.01, ***P < 0.001 compared with the Sham group; #*P* < 0.05, ##*P* < 0.01 compared with the Model group.

The apoptosis rate was significantly increased on days 7, 14, and 28 in the Model group (*P* < 0.001, *P* < 0.01, respectively; Figure 1). After rehabilitation training, except for day 7, the brain injury rate in the RT group was significantly inhibited on days 14 and 28 (*P* < 0.05 or *P* < 0.01, respectively; as shown in Figure 1). This result shows that, after rehabilitation training was provided for the CCI rats for 14 days, their brain nerve injuries were obviously controlled and were significantly improved after 28 days.

### 3.2 Effect of rehabilitation training on Notch1 in brain tissues

**Figure 2:**
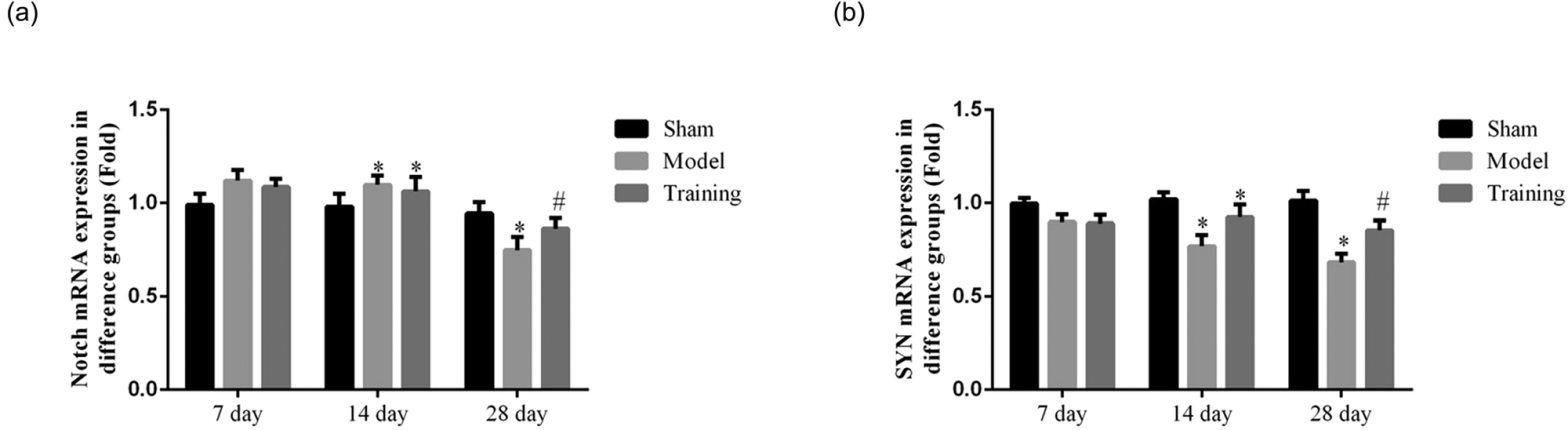
The Notch and SYN mRNA expressions in different groups by RT-qPCR assay. Sham: Sham group; Model: nerve injuries in CCI rat model; Training: nerve injuries in CCI model rats that were treated with rehabilitation training exercises. (a) The Notch mRNA expression in different groups; *P < 0.05 compared with the Sham group; #*P* < 0.05 compared with the Model group. (b) The SYN mRNA expression in different groups; *P < 0.05 compared with the Sham group; #*P* < 0.05 compared with the Model group.

**Figure 3:**
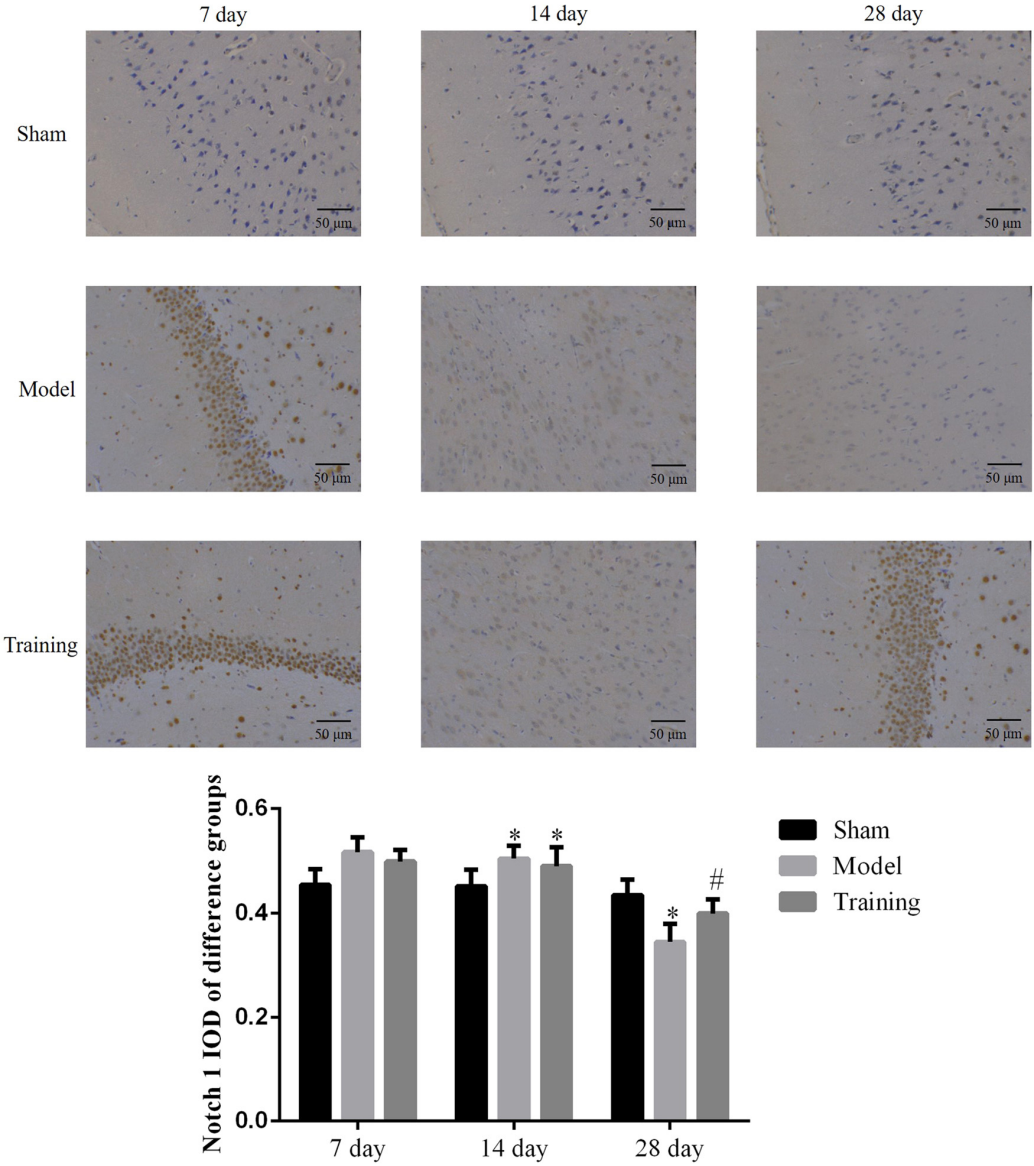
The Notch protein expression of different groups by IHC assay (×200). Sham: Sham group; Model: nerve injuries in CCI rat model; Training: nerve injuries in CCI model rats that were treated with rehabilitation training exercises; *P < 0.05 compared with the Sham group; #*P* < 0.05 compared with the Model group.

RT-qPCR was used to detect the Notch1 level in the brain tissue, and results show that the Notch1 gene level of the brain tissue of rats did not change significantly on days 7 and 14. On day 28, the Notch1 mRNA level in the brain tissue was significantly lower in the Model group (*P* < 0.05), while the Notch1 level in the RT group was higher than that of the Model group but lower than that of the Sham group (*P* < 0.05, respectively; as shown in Figure 2a). IHC was used to detect the Notch1 level in the brain tissue, and the data show that the Notchl level in the brain tissue of rats in different groups did not change significantly on days 7 and 14. On day 28, the expression level of Notchl was lower in the Model group than in the Sham group (*P* < 0.05, Figure 3), while there was no significant difference between the RT and Sham groups. For details, see Figures 2a and 3.

### 3.3 Effect of rehabilitation training on SYN in brain tissues

**Figure 4:**
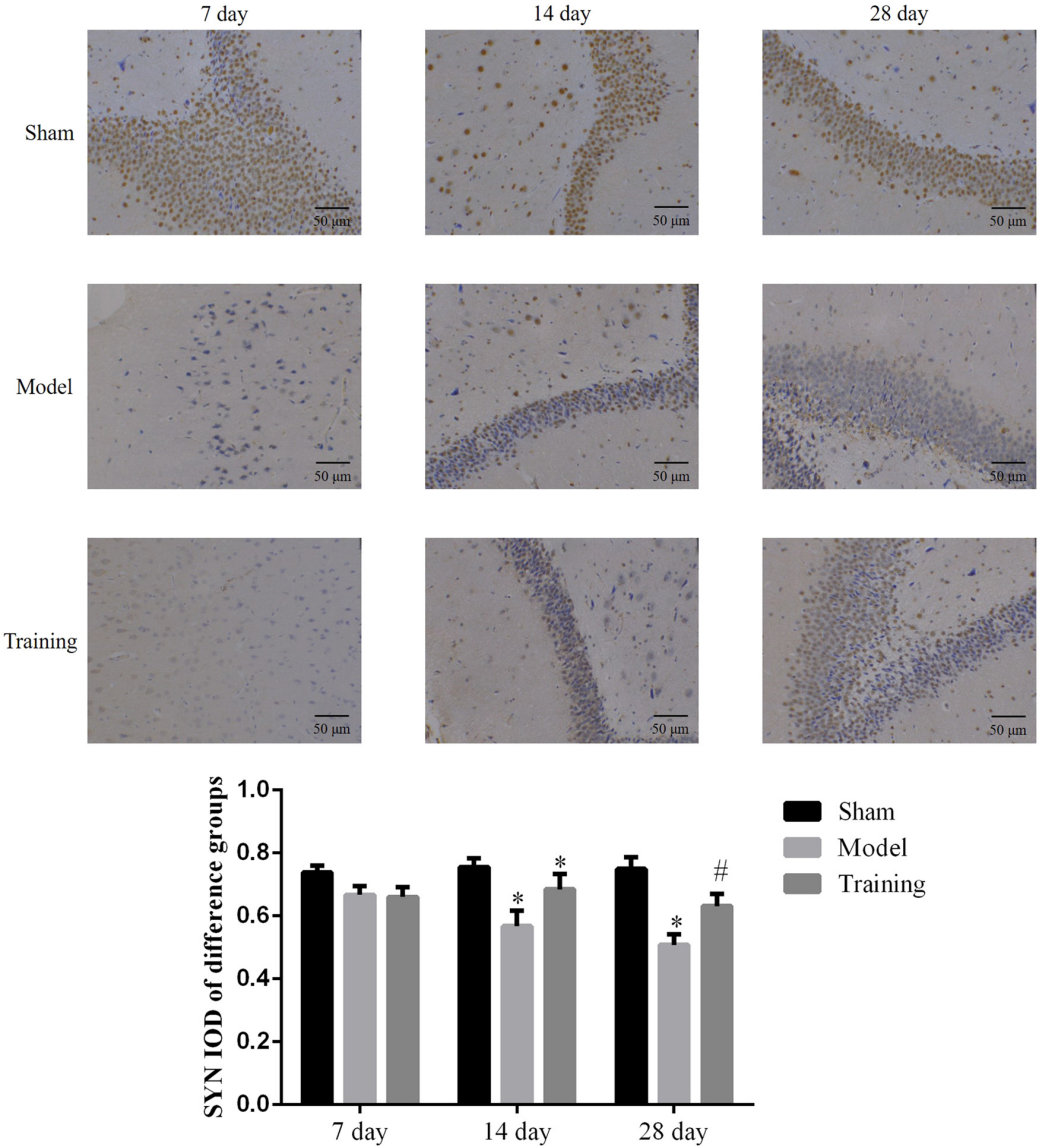
The SYN protein expression of different groups by IHC assay (×200). Sham: Sham group; Model: nerve injuries in CCI rat model; Training: nerve injuries in CCI model rats that were treated with rehabilitation training exercises; *P < 0.05 compared with the Sham group; #*P* < 0.05 compared with the Model group.

SYN mRNA level in the brain tissue was detected by RT-qPCR, and results show that the SYN mRNA level in the hippocampus in all groups did not change significantly on day 7. On day 14, the gene expression level of SYN in the RT and Model groups were lower (*P* < 0.05, Figure 2b). On day 28, the gene expression level was lower in the Model group (*P* < 0.05, Figure 2b), while the gene expression level in the RT group was lower (*P* < 0.05) but higher than that of the Model group (*P* < 0.05, Figure 2b). IHC was used to detect the SYN level in the brain tissue, and results show that the SYN level in all groups did not change significantly on day 7. On day 14, the SYN mRNA level was lower in the Model group (*P* < 0.05), while the RT group had no significant difference with both the Sham and Model groups. On day 28, the expression level in the Model group was lower than that in the Sham group (*P* < 0.05), the expression level in the RT group was higher than that in the Model group (*P* < 0.05), and the Sham group had no significant differences from the RT group. For details, see Figures 2b and 4.

## 4 Discussion

Rehabilitation training has been used in clinical rehabilitation for years and can effectively improve the motor functions of patients. The Notch pathway is a classical and conservative pathway that regulates development, and its association with the injury mechanism of cerebral ischemia has drawn increasing attention. The pathway members have been reported in research on ischemic injury, reperfusion after ischemia, and regulation of stem cells. The Notch pathway plays a major role in the morphology of neurons and neural stem cells [12,13,14]. Among the Notch pathway members, the receptor Notch 1 has been shown to be expressed in the hippocampus domain of the adult brain, affecting the neural development of the hippocampus, and to be involved in the processes of angiogenesis and neurogenesis after cerebral ischemia injury [15]. The absence of Notchl in the brain tissue may separate the long-term potentiation (LTP) and long-term depression (LTD), resulting in the decreased or even lost abilities of learning and short-term memory [16]. The Notch signaling pathway plays a major role in enhancing LTP, improving the learning and memory abilities, and alleviating brain damages [16,17]. In this study, apoptosis and Notch1 expression in the brain tissue of CCI rats were detected, and the results show that the damages and Notch1 expression in the brain tissue changed with the prolongation of the cerebral ischemia in the Model and RT groups. With the prolongation of the condition, the brain tissue damages in rats could be healed to some extent. Moreover, it shows that the recovery in the RT group was significantly better than that in the Model group. Meanwhile, on days 7 and 14, the Notchl contents in the Model and RT groups were higher than that in the Sham group. A possible reason might be that the Notch signaling pathway activation induced by CCI could have led to a compensatory increase in Notchl level, so as to compensate for the ischemia-induced neuronal loss. With the prolongation of the condition, the Notch pathway activation gradually weakened and even seemed inhibited. As a result, on day 28, the Notchl decreased significantly in the Model group. Although the RT group also showed a downward trend, its level was still significantly higher than that in the Model group, suggesting that the increase in Notchl on day 28 might be related to rehabilitation training.

SYN can better reflect the synaptic plasticity of the nervous system against the ischemic injury. As a transmembrane protein of synaptic vesicles, SYN is an essential structural protein that links nerve repair and interneuron communication and is related to the structure and functions of synapses. Its changes are often used as an indicator of the repair of the brain tissue [18]. Studies have shown that rehabilitation training can improve SYN in the hippocampus, enhance LTP, and strengthen the learning and memory abilities [19]. The results of this study were consistent with previous findings. It was observed that rehabilitation training could increase the expression of SYN in the hippocampus of CCI rats. On day 28 of training, the SYN level was increased significantly more in the RT group than in the Model group. After CCI, the expression level of SYN in the hippocampus decreased, while a certain period of rehabilitation training could increase the expression of SYN.

In summary, rehabilitation training could effectively enhance the recovery of brain injury, and its underlying mechanism might be related to improving the expressions of Notchl and SYN in the brain tissue. Many studies and our previous experiments have confirmed that rehabilitation training can improve the learning and memory abilities of rats. So, does it work by affecting the changes of Notchl and SYN? There are complex pathophysiological changes in the body after CCI. It was found that after 28 days of rehabilitation training, the Notchl level in the hippocampus was higher than that in the Model group, suggesting that rehabilitation training might activate the Notch pathway, leading to the changes in Notchl. However, the expression of Notchl is influenced and regulated by many factors, and the underlying mechanism of the Notch pathway affecting the learning and memory functions still remains unclear. In addition, the specific mechanism by which the receptor Notchl regulates learning and memory functions is also not fully understood. For this reason, our further study should be designed to observe the changes of pathway-related ligands, target genes, and regulatory factors. As changes in the content of Notchl vary at different time points, the training time should be extended in the subsequent study so as to thoroughly observe the changes of Notchl.
